# Outbreak of NDM-5-producing *Klebsiella pneumoniae* ST307: an emerging high-risk antimicrobial resistance clone in Shanghai, China

**DOI:** 10.1128/msystems.01369-23

**Published:** 2024-03-20

**Authors:** Junying Zhu, Guangyu Wang, Min Li

**Affiliations:** 1 Department of Laboratory Medicine, Renji Hospital, School of Medicine, Shanghai Jiao Tong University, Shanghai, China; 2 Faculty of Medical Laboratory Science, College of Health Science and Technology, School of Medicine, Shanghai Jiao Tong University School of Medicine, Shanghai, China; 3 Department of Cardiology, Renji Hospital, School of Medicine, Shanghai Jiao Tong University, Shanghai, China; Third Institute of Oceanography Ministry of Natural Resources, Xiamen, China

**Keywords:** carbapenem resistance, *Klebsiella pneumoniae*, outbreak, ST307 high-risk clone

## Abstract

The high-risk clone *Klebsiella pneumoniae* ST307, associated with various carbapenem resistance genes, exhibits a global distribution and prevalence. However, in China, it has remained sporadic and has rarely been detected. In this study, we reported an outbreak caused by nine ST307 CRKP isolates harboring *bla*_NDM-5_ in Shanghai, China, in 2022. We employed antimicrobial susceptibility testing, conjugation assay, whole-genome sequencing (WGS) and comparative genomics, phylogenetic analysis, and fitness and virulence comparison to further characterize the isolates causing the outbreak. Besides *bla*_NDM-5_, these nine isolates co-carried *bla*_CTX-M-15_ and *bla*_DHA-1_, exhibiting nearly identical resistance profiles with high-level resistance to carbapenems and ceftazidime/avibactam, while showing susceptibility to colistin and tigecycline. *bla*_NDM-5_ was located on an IncX3 plasmid of 45,403 bp with a high frequency of conjugative ability. Phylogenetic and single-nucleotide polymorphism (SNP) analysis indicated the nature of clonal transmission with a maximum of five SNPs between these nine isolates, and they were closely related to strains obtained from the United States. ST307 isolates in our study showed a relatively lower virulence but higher growth rates and certain adaptability compared with ST11 isolates. Clinical investigation revealed that shared nursing staff in a mixed emergency intensive care unit ward and doctors’ movement between wards might be responsible for the outbreak. The nonexistence before and sudden emergence of ST307 suggested that the currently circulating ST307 clone was a newly introduced superbug in our hospital. In conclusion, we revealed that *bla*_NDM-5_-producing ST307 CRKP isolates, a globally significant high-risk clone, are spreading in China, posing a substantial threat to public health.

**IMPORTANCE:**

The high-risk clone ST307, associated with various carbapenemases, including KPC, NDM, and OXA, has a global distribution. However, it is rarely reported in China, let alone causing outbreaks. Here, we found an outbreak caused by the clonal transmission of nine ST307 CRKP isolates. Clinical investigation revealed that shared nurses in a mixed emergency intensive care unit ward and doctors’ movement between wards might be responsible for the outbreak. In our study, the nine NDM-5-producing ST307 isolates exhibited high-level resistance to carbapenems and ceftazidime-avibactam, high conjugative ability to *Escherichia coli* J53, and certain adaptability to environment, phylogenetically closet to the United States. All these features make ST307 clone the next successful clone comparable to ST11 clone in China. Therefore, it is imperative for us to vigilantly monitor the prevalence of carbapenem-resistant *Klebsiella pneumoniae* and promptly implement measures to control the spread of *K. pneumoniae* ST307 in China.

## INTRODUCTION

Carbapenem-resistant *Klebsiella pneumoniae* (CRKP) has been a significant global public health issue for years and is increasingly becoming a serious crisis in clinical settings, particularly in intensive care units (ICUs) (1, 2). The strong adaptability to diverse and challenging environments has contributed to the worldwide prevalence and distribution of CRKP (3, 4). Additionally, high-risk clones carrying antimicrobial resistance (AMR) genes also play a crucial role in the intercountry and interregional spread of CRKP. The endemic sequence type (ST) 258 and ST512 in the United States and the dominant ST11 in China are typical representatives of successful clones mediating the dissemination of carbapenem resistance determinants (5, 6).

Moreover, an increasing number of newer epidemic clones join in the dissemination of AMR genes (7). An example is the *K. pneumoniae* ST307 clone, first detected in the Netherlands in 2008 and rapidly became epidemic in Italy, Colombia, the United States (Texas), and South Africa (7–9). Although ST307 has been globally associated with various carbapenemases, there are still regional characteristics in the carbapenemase-producing types to some extent. ST307 CRKP is mainly OXA-181 producing in South Africa, OXA-48 producing in Spain, and KPC-2 producing in Italy, respectively (9–11). In China, ST307 CRKP strains have remained sporadic and have rarely been detected. A recent study found eight ST307 isolates collected from the blood of patients with bloodstream infections in nine Chinese hospitals and identified their association with small nosocomial transmission (12). In addition, IMP-38-producing ST307 *K. pneumoniae* strains from neonatal unit were also reported in China (13). However, the ST307 *K. pneumoniae* clone is rarely associated with the production of NDM-5 in China. Evidence of virulence and fitness between ST307 and other clones is also currently lacking. In this study, we described an outbreak caused by NDM-5*-*producing ST307 CRKP in a teaching hospital in Shanghai, China. Furthermore, we analyzed the molecular characteristics of ST307 isolates and compared the virulence and fitness between ST307 and ST11 clones.

## RESULTS

### Hospital settings and outbreak description

**Fig 1:**
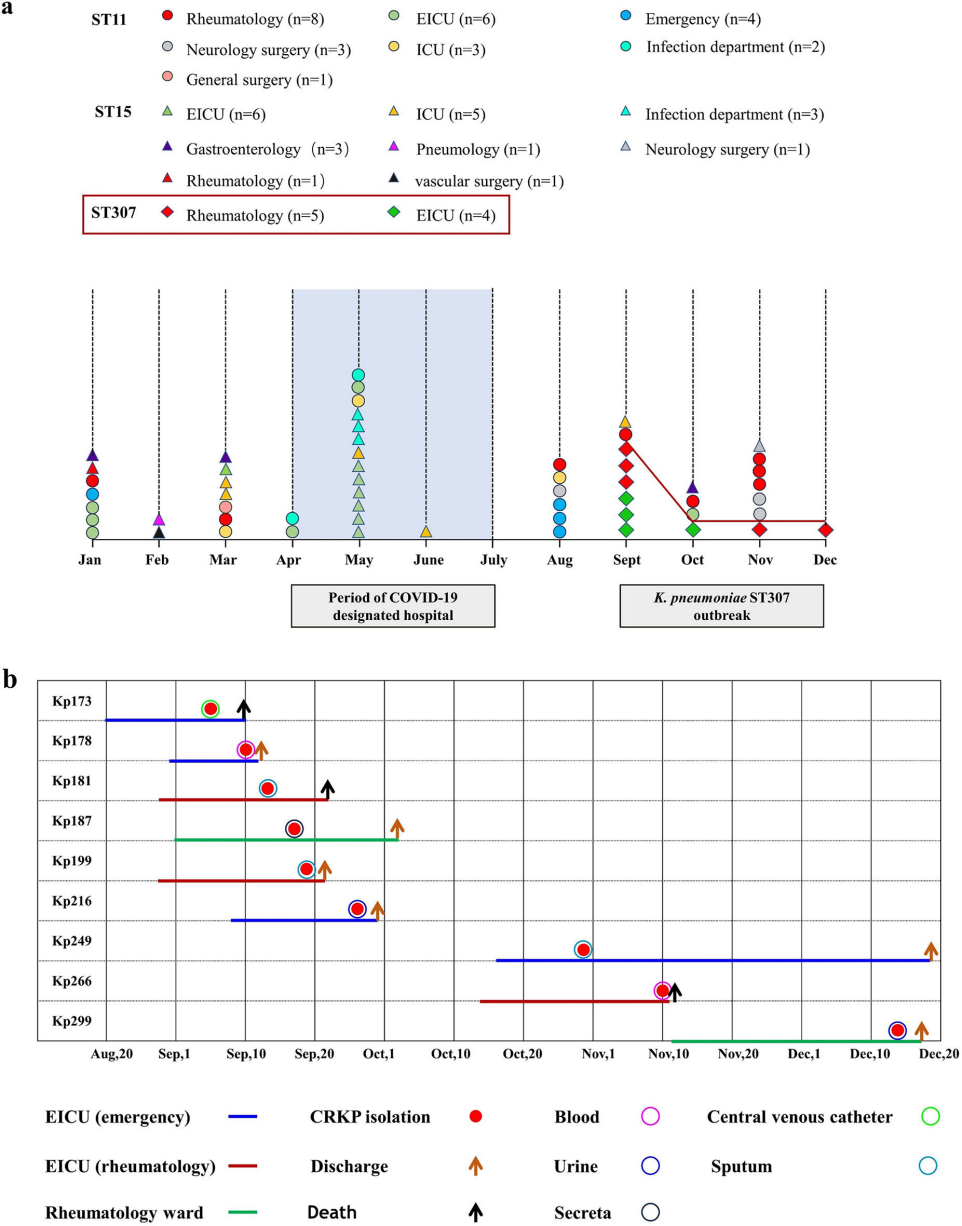
Features of CRKP isolates in SRJH throughout 2022. (**a**) Department distribution and isolation timeframe of 57 CRKP isolates, including ST11 (*n* = 27), ST15 (*n* = 21), and ST307 (*n* = 9). Department is represented by different colors, and STs are shown in different shapes. Isolates below the red line between September and December represent the ST307 outbreak isolates. (**b**) Time table and clinical information of the ST307 outbreak cases.

A total of 59 clinical CRKP isolates were collected in the south branch of Renji Hospital (SRJH) throughout 2022. The 59 isolates were obtained from a variety of specimens, including respiratory specimens (*n* = 30), urine (*n* = 12), blood (*n* = 10), intra-abdominal fluid (*n* = 3), central venous catheter (CVC) (*n* = 2), secreta (*n* = 1), and soft tissues (*n* = 1). The majority of clinical isolates (27.1%,16/59) were cultured from the emergency ICU (EICU), followed by the rheumatology ward (23.7%, 14/59). Whole-genome sequencing (WGS) was used to identify STs, and those isolates were belonged to ST11 (45.8%, 27/59), ST15 (35.6%, 21/59), ST307 (15.2%, 9/59), ST1737 (1/59, 1.7%), and ST3691 (1/59, 1.7%) (Fig. 1a).

As indicated in Fig. 1a, the predominant STs circulated in SRJH underwent a significant change over the course of 2022. Before April, ST11 and ST15 were the primary epidemic clones, consistently detectable in most departments. In April, Shanghai, China, experienced a wave of COVID-19 pandemic, and SRJH was designated as the hospital specifically for patients with severe COVID-19 infections. During this period, ST15 was the predominant clone, accounting for 68.7% (11/16) among COVID-19 patients with CRKP coinfections. In July, the wave of COVID-19 pandemic was under control, and SRJH was no longer designated as a hospital specifically for COVID-19 patients. After implementing comprehensive and strict disinfection measures against residual COVID-19 and possible pathogenic bacteria, SRJH reopened for patient admissions. Between September and December, we notably observed the detection of ST307 isolates, a clone never detected in our hospital, among 45% (9/20) of CRKP isolates, which indicated the possibility of an outbreak (Fig. 1a).

The first ST307 CRKP strain (Kp173) was isolated from the sputum of a patient with a 15-day history of fever after chemotherapy in 5 September 2022. The patient had a medical history of NK/T-cell lymphoma of the nasal cavity and was admitted to EICU for severe pneumonia. After 16-day hospitalization, a ST307 CRKP strain was detected from CVC of the patient. Notably, 40% (8/20) of the beds in EICU are dedicated to patients with critically ill rheumatic diseases, and 60% (12/20) of the beds are reserved for severe emergency patients according to hospital bed allocation. Consequently, we detected six additional ST307 CRKP isolates from three patients with severe rheumatic and three emergency patients in the same EICU ward. During the same period, we also identified two ST307 isolates from two patients in the general rheumatology ward (Fig. 1b).

The median age of the 9 patients (5 males and 4 females) was 70 years (range, 41–86 years). These patients had various underlying diseases, including tumors, heart failure, dermatomyositis, temporal arteritis, interstitial lung disease, diabetes, adult-onset Still’s disease, and systemic lupus erythematosus. Most patients (77.8%, 7/9) had received carbapenem antimicrobial treatments, including meropenem and/or imipenem. Except for one patient, the other eight patients all underwent invasive procedures involving medical devices, including CVC, tracheal intubation, and the like. In the end, two patients experienced deteriorating health conditions during discharge and three patients died of CRKP infection (Table S1).

### Antimicrobial susceptibility testing, resistance determinants profile

Susceptibility testing showed that the nine isolates had almost identical antibacterial susceptibility profiles. All the isolates exhibited high-level resistance to CAZ/AVI (MIC, ≥128 µg/mL), imipenem (MIC, ≥64 µg/mL), meropenem (MIC, ≥64 µg/mL), ceftazidime (MIC, ≥64 µg/mL), cefepime (MIC, ≥32 µg/mL), aztreonam (MIC, ≥64 µg/mL), cefoperazone/sulbactam (MIC range, ≥64 to ≥128 µg/mL), and levofloxacin (MIC, 8 µg/mL) while remaining susceptible to colistin (MIC, 0.5 µg/mL) and tigecycline (MIC range, 0.5–1 µg/mL). Moreover, 66.6% (6/9) isolates showed resistance to amikacin (MIC, ≥64 µg/mL) and 88.9% (8/9) isolates showed resistance to gentamicin (MIC, 16 µg/mL) (Table 1).

**TABLE 1:** K-locus, multi-locus sequence typing, and antimicrobial susceptibility results of the nine ST307 NDM-5-producing CRKP isolates^*a*^

Isolates	K/O type	ST type	Minimum inhibitory concentration (μg/mL)											
			AMK	GEN	LEV	CZA	FEP	SCF	COL	CAZ	IPM	MEM	ATM	TGC
Kp173	KL102/O2	ST307	≥64	16	8	≥128	≥32	≥64	0.5	≥64	≥64	≥64	≥64	0.5
Kp178	KL102/O2	ST307	≥64	16	8	≥128	≥32	≥128	0.5	≥64	≥64	≥64	≥64	1
Kp181	KL102/O2	ST307	4	16	8	≥128	≥32	≥128	0.5	≥64	≥64	≥64	≥64	1
Kp187	KL102/O2	ST307	≥64	16	8	≥128	≥32	≥128	0.5	≥64	≥64	≥64	≥64	0.5
Kp199	KL102/O2	ST307	≥64	16	8	≥128	≥32	≥64	0.5	≥64	≥64	≥64	≥64	0.5
Kp216	KL102/O2	ST307	4	16	8	≥128	≥32	≥64	0.5	≥64	≥64	≥64	≥64	0.5
Kp249	KL102/O2	ST307	4	2	8	≥128	≥32	≥128	0.5	≥64	≥64	≥64	≥64	0.5
Kp266	KL102/O2	ST307	≥64	16	8	≥128	≥32	≥64	0.5	≥64	≥64	≥64	≥64	0.5
Kp299	KL102/O2	ST307	≥64	16	8	≥128	≥32	≥128	0.5	≥64	≥64	≥64	≥64	0.5

^*a*^ AMK, amikacin; GEN, gentamicin; LEV, levofloxacin; CZA, ceftazidime/avibactam; FEP, cefepime; SCF, cefoperazone/sulbactam; COL, colistin; CAZ, ceftazidime; IPM, imipenem; MEM, meropenem; ATM, aztreonam; TGC, tigecycline.

WGS data analysis revealed that all nine isolates belonged to ST307 KL102 and O2 locus. Resistance genes profiling revealed the presence of the chromosomal *bla*_SHV-28_ and a gene encoding a truncated OmpK35 in all nine isolates. Concerning the acquired resistance genes, along with the *bla*_NDM-5_, *bla*_CTX-M-15_, and *bla*_DHA-1_ β-lactamase genes, detected in all isolates, other determinants associated with resistance to β-lactam antibiotics (*bla*_LAP-2_), aminoglycosides (*armA*, *strA*, *strB*), sulfonamides (*sul1*, *sul2*), trimethoprim (*dfrA14*), and quinolones (*qnrB1*, *qnrB4*, *qnrS1*) were also present in all nine isolates. No virulence genes were detected in any of the nine outbreak isolates.

### Phylogenetic relatedness

To elucidate the clonal relatedness among the nine NDM-5-producing ST307 strains, we mapped the nine ST307 genomes to the reference genome Kp178 which was sequenced using the PacBio platform. Paired distance was used to define the clonal relatedness of the isolates. The analysis revealed a maximum of five single-nucleotide polymorphisms (SNPs) (minimum, 0) between the nine isolates, strongly indicating clonal transmission (Fig. S1).

**Fig 2:**
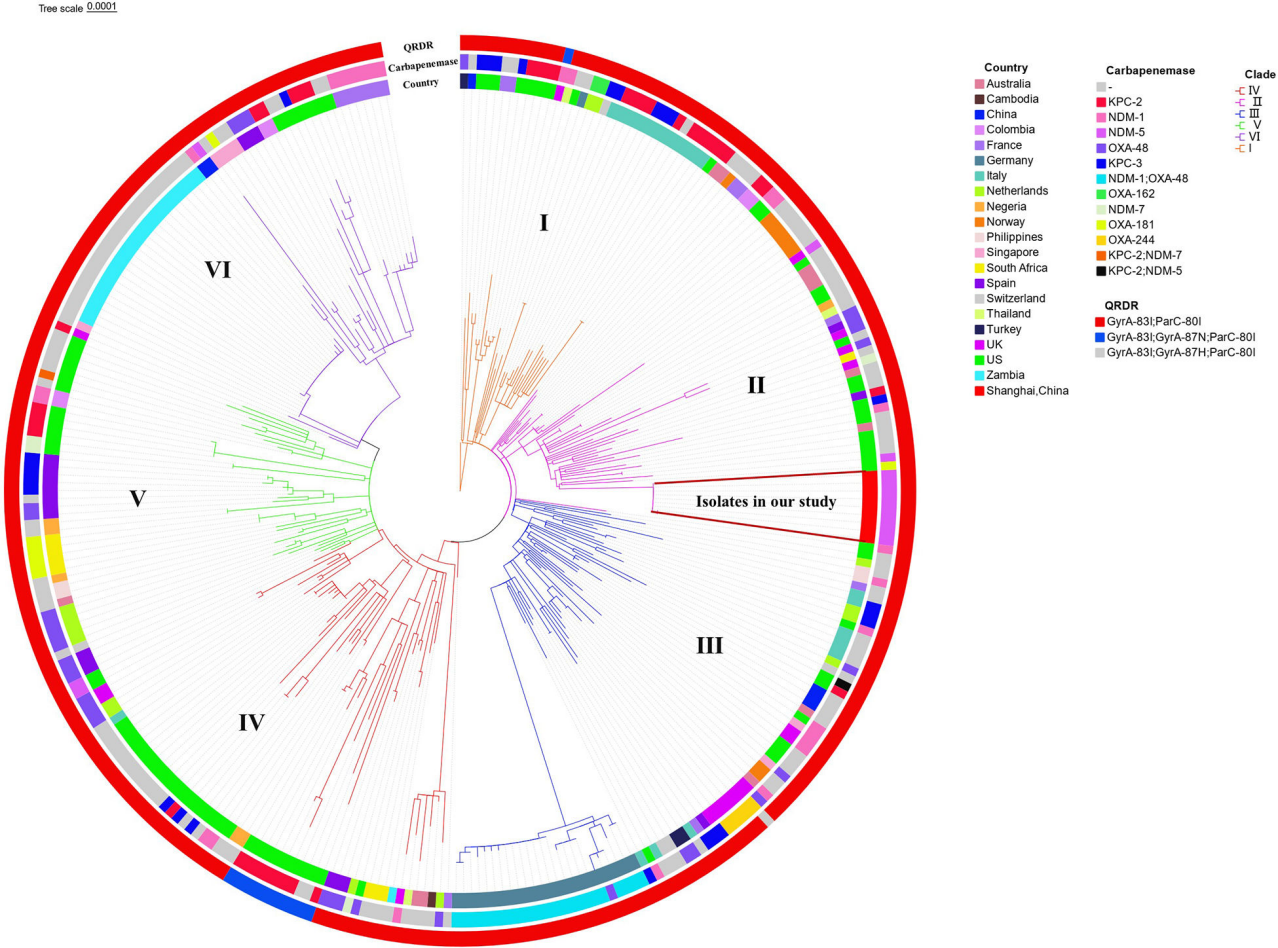
Maximum-likelihood tree of *K. pneumoniae* ST307 isolates. The ST307 genomes included nine from this study and 304 internationally representative isolates from 20 countries (downloaded from NCBI SRA database).

When we compared the nine outbreak isolates with 304 global isolates, the phylogenetic tree divided the 313 ST307 isolates into six distinct clades: clades I, II, III, IV, V, and VI. Our nine NDM-5-producing outbreak strains were grouped into clade II and were phylogenetically closet to strains obtained from the United States. Similar to global ST307 isolates with 90% (274/304) containing *bla*_CTX-M-15_, all our nine isolates carried *bla*_CTX-M-15_. In contrast, all nine isolates in our study carried *bla*_DHA-1_, while only 4.9% (15/304) of global ST307 isolates carried this gene. Furthermore, sequence analysis of the mutations in the quinolone resistance-determining regions (QRDR) in chromosomal gyrA and parC indicated that all the 313 isolates contained the ParC-80I and GyrA-83I mutations, and 13 global ST307 isolates contained additional GyrA-87N or GyrA-87H mutations (Fig. 2).

### Genome and molecular characteristics of Kp178

Kp178 was selected for further sequencing using the PacBio platform. The genome size was 5,956,808 bp, comprising a 5,375,566 bp chromosome, 6 circular plasmids, and 1 linear plasmid. Among the seven plasmids are *bla*_NDM-5_ harboring plasmid (pKp178-NDM-5), *bla*_CTX-M-15_ harboring plasmid (pKp178-CTX-M-15), *bla*_DHA-1_ harboring plasmid (pKp178-DHA-1), *qnrB1* harboring plasmid (pKp178-qnrB1), and three plasmids devoid of resistance or virulence genes. pKp178-NDM-5 was an IncX3 type plasmid of 45,403 bp carrying only *bla*_NDM-5_ resistance genes. It could be transferred to *Escherichia coli* J53 through a conjugation assay at a frequency of 10^−4^ transconjugants per donor cell. Comparative genomics showed that pKp178-NDM-5 displayed 100% coverage and 100% identity to pHD8763-NDM (GenBank accession no. ON209135.1). pKp178-DHA-1 was a 206,048 bp plasmid containing only *bla*_DHA-1_. Besides *bla*_CTX-M-15_, pKp178-CTX-M-15 also carried *bla*_LAP-2_, *qnrS1,* and *dfrA14*. Likewise, *sul2* and *tet*(*A*) were also present on the *qnrB1* harboring pKp178-qnrB1. In terms of virulence genes, no typical virulence factors or integrative conjugative elements (ICEKp) were detected on plasmids or the chromosome (Table S2).

### Comparison of adaptability to environment and virulence between ST307 and ST11 isolates

**Fig 3:**
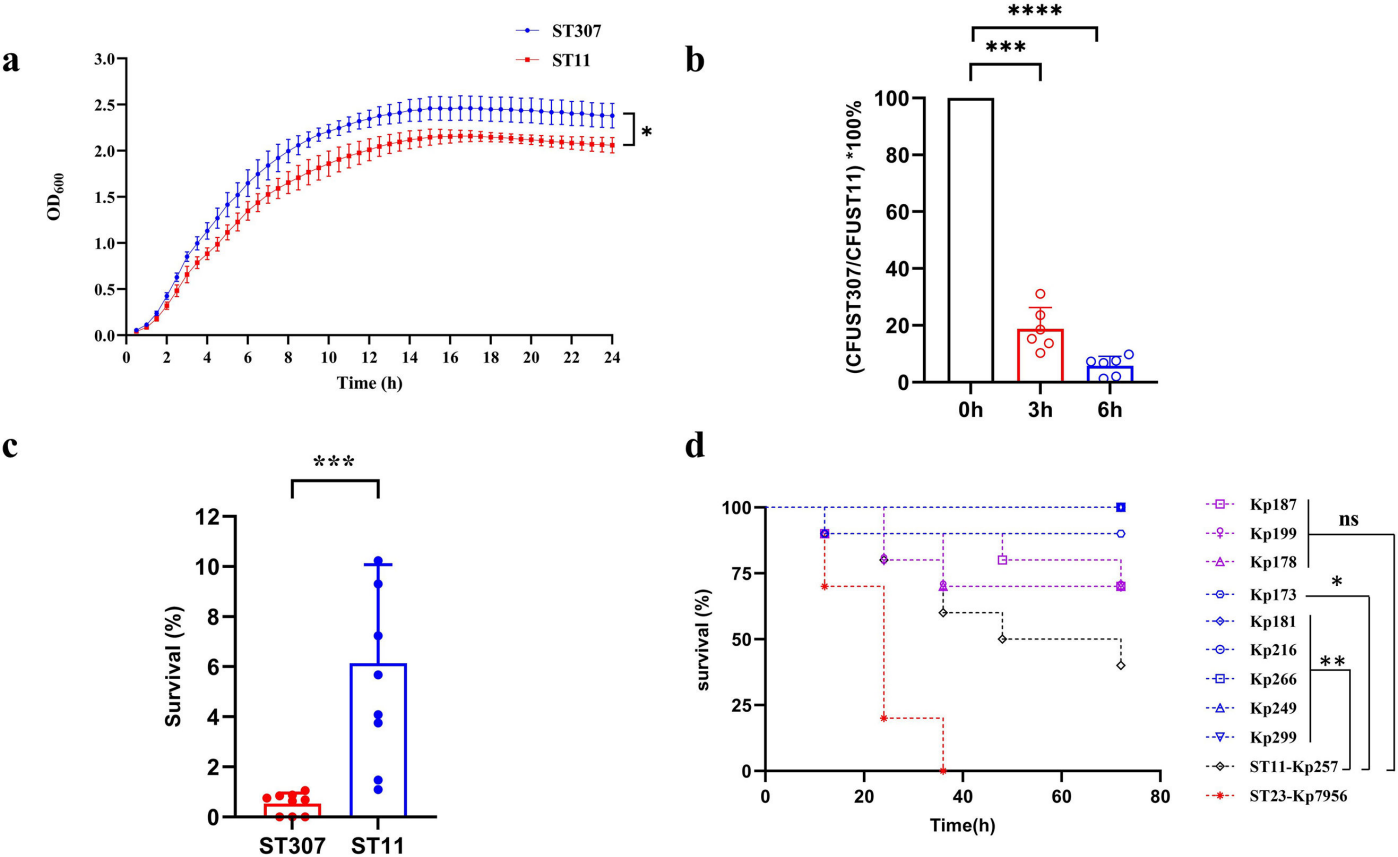
Comparison of adaptability and virulence between ST307 and ST11 isolates in our study. (**a**) Growth curve of ST307 and ST11 isolates. (**b**) *In vitro* competition experiments between ST307 and ST11 isolates. (**c**) Serum resistance of ST307 and ST11 isolates. Survival (%) indicated the proportion of CFU at 2 and 0 h. (**d**) Survival curves for *G. mellonella* infection model. *P < 0.05; ***P < 0.001; ****P < 0.0001.

Growth curve assays showed that the growth rate of ST307 isolates was significantly higher than that of ST11 isolates at almost all time point from the 2nd to the 24th hour (Fig. 3a). However, *in vitro* competition assays, the ratio of colony-forming units (CFU) between ST307 and ST11 isolates exhibited a significant decrease after the two clones were co-cultured for 3 and 6 h. Although the result is influenced by distinct genetic backgrounds of ST307 and ST11 clones, it suggests, to a certain extent, the impaired growth of ST307 isolates when cultured with ST11 isolates (Fig. 3b). Furthermore, in the presence of pooled human serum, all nine ST307 isolates exhibited sensitivity while ST11 showed intermediate sensitivity to serum kill (Fig. 3c). In the *Galleria mellonella* infection model, Kp257, an ST11 CRKP, was selected for the comparison of virulence with ST307 isolates. As shown in Fig. 3d, the mortality of *G. mellonella* infected by Kp257 was significantly higher than that of those infected with six ST307 isolates (Kp173, Kp181, Kp216, Kp249, Kp266, Kp299), while there was no statistical difference when compared with *G. mellonella* infected by Kp178, Kp187, and Kp199 (Fig. 3d). Altogether, these results suggested that NDM-5-producing ST307 isolates in our outbreak showed a relatively low virulence, whereas they exhibited higher growth rate and certain adaptability to environment.

## DISCUSSION

Tracking the emergence of high-risk clones during an outbreak and understanding their dissemination path are crucial for monitoring and controlling the spread of CRKP. In this study, we report an outbreak caused by NDM-5-producing ST307 CRKP in the rheumatology and EICU wards of a teaching hospital. In our hospital, the EICU was a mixed ward, with 40% of the beds allocated for patients with severe rheumatic diseases and 60% for those with severe emergency patients. The sharing of nurses among patients in the mixed EICU and the relatively mobile movement of rheumatology doctors between EICU and rheumatology wards might be responsible for the dissemination of ST307 in these two wards. Additionally, ST11 and ST15 were the predominant clones circulating in our hospital before and during the wave of COVID-19 pandemic, while ST307 clone was not detected until September 2022. Based on this observation, we speculated that the ST307 clone was newly introduced to our hospital. Moreover, we have recently detected the emergence of ST307 CRKP isolate in the west branch of Renji Hospital, which indicated the possibility inter-branch spread of ST307 clone.

SNP and phylogenetic analysis revealed that the clonal spread of *K. pneumoniae* ST307, a recognized high-risk clone harboring various carbapenemases and mediating nosocomial dissemination worldwide, was the cause of this outbreak. Large-scale outbreak sustained by the ST307 clone in South Africa and Germany has also been reported (9, 14). More surprisingly, ST307 is replacing ST258 as the most prevalent clone associated with multidrug resistance genes in Texas and Colombia (8, 15). These findings, altogether, indicate the success of ST307 lineage in spreading antimicrobial resistance genes. In China, however, only a few sporadic reports about the ST307 lineage have been reported. Our study suggests that the ST307 clone might be overlooked and spreading underground. Phylogenetic relatedness revealed that our nine ST307 outbreak strains were closet to strains obtained from the United States which explain the origin of ST307 isolates in SRJH to some extent. This indicates that ST307 has the ability to be introduced to other countries and cause devastating country-wide outbreaks associated with substantial healthcare costs.

Another manifestation of ST307 as a successful clone is its ability to carry a variety of carbapenemases. Besides KPC-2, several studies have reported ST307 CRKP also carried novel KPC variants (KPC-62, KPC-39, KPC-46, KPC-66, and KPC-92) which exhibited CAZ/AVI resistance (16–18). Similarly, NDM-5 can also hydrolyze CAZ/AVI, leaving few treatment options for infections, especially when isolates co-produce CTX-M and DHA-1 (19). In our study, two patients had deteriorating health conditions during discharge, and three patients died of CRKP infection. The high morbidity and mortality due to severe infection and antimicrobial resistance indicated that NDM-5-producing ST307 CRKP infection is a severe situation and needs more attention.

In the genome of Kp178, *bla*_NDM-5_ was located on a 45,403 bp IncX3 plasmid with high conjugation and transfer capabilities. It has been reported that China is the country with the most prevalent IncX3 plasmid, and *K. pneumoniae* ST307, ST11, and ST15 are the most popular types among IncX3 plasmid-containing isolates (20). As a typical self-transfer plasmid, IncX3 plasmids have high conjugation ability facilitating the dissemination of resistance genes, as observed in our study. Furthermore, IncX3 plasmids are frequently associated with *bla*_NDM_ and its variants, followed by *bla*_OXA-181_ and *bla*_KPC_. However, in China, *bla*_NDM-5_ was the most gene carried in IncX3 plasmids, while *bla*_KPC_-harboring IncX3 plasmids have not been reported.

When compared with *bla*_KPC-2_-harboring ST11 isolate, the ST307 clone in our study showed lower virulence in the *G. mellonella* infection model and exhibited sensitivity to human serum. Despite ST11 isolates exhibiting a growth advantage over ST307 isolates when competing for space and nutrients in the same environment by *in vitro* competition test, ST307 grew significantly faster than ST11 when cultured alone, which indicated the certain environmental adaptability of ST307 clone. Hence, it is imperative to monitor the epidemiology and evolution of the high-risk ST307 clone.

Although our study provided evidence about the emergence of the ST307 NDM-5-producing CRKP in China and compared the fitness, adaption, and virulence between ST307 and ST11 clones, it still has some limitations. First, partially representative assemblies from NCBI SRA database have been selected to construct the phylogenetic tree. Second, we did not use a more accurate mouse infection model to assess the virulence of ST307 isolates. In addition, long-term and large-scale surveillance is still needed to characterize the prevalence and evolution of ST307 CRKP in China.

In conclusion, we used a combination of WGS and epidemiologic techniques to identify a healthcare-associated outbreak caused by NDM-5 producing ST307 CRKP. The transmission of ST307 in our hospital might be attributed to shared nursing workers in a ward and relatively mobile doctors between wards. To sustainably reduce the spread of AMR-related pathogens, optimization of patients’ responsibility management, implementation of strict infection prevention and control measures, and effective disinfection and isolation measures are needed.

## MATERIALS AND METHODS

### Source of bacterial strains and hospital settings

We collected CRKP strains from SRJH between 1 January and 31 December 2022. Renji Hospital comprises a total of four branches, with SRJH located in the south of Shanghai. SRJH has 700 beds and serves patients nationwide, mainly due to the famous department of rheumatology.

In late February 2022, Shanghai, China, had experienced a wave of COVID-19 pandemic. SRJH was designated specifically for patients with severe COVID-19 infections between April and June. After the discharge of the last COVID-19 patient, we implemented comprehensive and strict disinfection measures against residual COVID-19 and possible pathogenic bacteria for 2 weeks. In July, our hospital reopened to admit patients. In 5 September, we identified the first ST307 CRKP isolate, a new clone that had never existed in our hospital before, circulating in the EICU department. Subsequently, additional eight ST307 CRKP strains were detected in EICU and rheumatology ward within 4 months indicating the possibility of an outbreak. Next, we primarily focused on the ST307 CRKP isolates and attempted to investigate the truth of the outbreak.

### Isolate identification, antimicrobial susceptibility testing, and conjugation experiment

Bacterial identity was confirmed using MALDI-TOF mass spectrometry (bioMérieux VITEK MS). Antimicrobial susceptibilities testing, including 12 different antibiotics, was determined using broth microdilution methodology and interpreted according to the guideline document M100-S31 established by the Clinical and Laboratory Standards Institute (21). ATCC 25922 was used as a quality control. A conjugation experiment was carried out with *E. coli* J53 as the recipient to determine the transferability of the *bla*_NDM-5_ gene, as described previously (22).

### Whole-genome sequencing and data analysis

Genomic DNA was extracted using TIANprep Midi Plasmid Kit and sequenced on the HiSeq X ten PE150 sequencer platform (Illumina, USA) with a 2 × 150 bp read length (Majorbio Bio-pharm Technology, Shanghai, China) as previously described (23). Kpn178 was further sequenced using the PacBio platform.

*De novo* assembly of the clean data was performed in CLC Genomics Workbench 12.0 (Qiagen) with the default options. The assembled contigs were used for BLAST at the drug-resistant gene database (Resfinder database, 2023-02) and at the Virulence Factors Database (VFDB, 2023-05) to confirm if the resistant or virulence genes were present. WGS data were utilized for multi-locus sequence typing and identification of K- and O-loci using online tools available at https://services.healthtech.dtu.dk/.

We compared the nine genomes from the outbreak sequenced in this study (deposited in the NCBI Bioproject database under accession no. PRJNA1012333) with 304 global ST307 genomes extracted from the NCBI SRA database. The global ST307 genomes included the United States (*n* = 91), the United Kingdom (*n* = 18), Zambia (*n* = 25), Spain (*n* = 20), Germany (*n* = 25), Italy (*n* = 24), the Netherlands (*n* = 15), France (*n* = 15), Australia (*n* = 12), South Africa (*n* = 9), China (*n* = 6), Thailand (*n* = 3), Norway (*n* = 9), Singapore (*n* = 7), Colombia (*n* = 6), Nigeria (*n* = 6), Switzerland (*n* = 5), Philippines (*n* = 4), Turkey (*n* = 3), and Cambodia (*n* = 1).

SNPs calling and phylogenetic analysis were conducted in CLC Genomics Workbench 12.0 with the default options. The genome Kp178 was used as the reference template for read mapping. All genomes were mapped to the reference genome. A maximum-likelihood tree, constructed in CLC Genomics Workbench 12.0 using the general time reversible (GTR) model of nucleotide substitution with among-site rate heterogeneity across four categories (GTR+Γ), was subsequently annotated using ChiPlot (24).

### Comparison of fitness and virulence between ST307 and ST11 isolates

To assess the fitness and adaptability of the ST307 isolates, we selected three ST307 isolates (Kp178, Kp216, and Kp299) and three ST11 isolates (Kp94, Kp144, and Kp257) from our study for growth curve and *in vitro* competition growth experiments. In addition, serum killing assay and *G. mellonella* model were used to compare the virulence between ST307 and ST11 isolates. The genetic background of the three ST11 isolated was listed in Table S3.

Growth curve assay and *in vitro* competition assays were performed as previously described (25). Briefly, isolates cultured overnight in Luria-Bertani (LB) broth were diluted to an OD_600_ of 0.01 and grown at 37°C with shaking (200 rpm). The culture cell density was determined every 0.5 h by measuring the OD_600_ (Thermo Fisher Scientific, Shanghai, China). For the *in vitro* competition assays, six isolates were separately cultured overnight in LB broth at 37°C. The bacteria were diluted to 0.5 × 10^6^ CFU/mL, and equal volumes of ST307 and ST11 isolates were combined and cultured at 37°C with shaking. NDM-5-producing ST307 isolates can grow on plates containing 32 µg/mL CAZ/AVI, whereas KPC-producing ST11 isolates cannot. At 3 and 6 h, aliquots of the mixed bacteria were serially diluted and plated on LB agar plates with or without CAZ/AVI. CFU were counted after 24 h incubation at 37°C. The competitive index (CI) was determined as follows: CI = (CFU_ST307_/CFU_ST11_) × 100%.

Serum killing assay was conducted as previously described (26). A volume of 0.05 mL 0.5 × 10^6^ CFU/mL bacteria at early-log-phase cells was added to 0.15 mL pooled human serum obtained from healthy volunteers. Viable counts were determined at 0 and 2 h of incubation at 37°C on Mueller-Hinton agar. A drop in CFU counts to 1% of the original mixture was considered sensitive, whereas survival of at least 90% of the organisms was considered resistant after 2 h incubation.

We employed the infection model of wax moth larvae (~300 mg *G*. *mellonella*) to compare the virulence of ST307 and ST11 isolates. Log-phase cultures were washed and adjusted to a concentration of 0.5 × 10^7^ CFU/mL with phosphate-buffered saline. *G. mellonella* larvae were obtained from Tianjin Huiyude Biotech Company (Tianjin, China) and distributed into experimental groups (10 larvae per group). Mortality rates were monitored for a period of 3 days. We selected one KPC-2-producing *K. pneumoniae* strain, Kp257, which belonged to ST11, to compare the virulence of ST307 and ST11 isolates. Additionally, one ST23 *K. pneumoniae* strain, Kp7956, isolated from the abscess drainage of a patient with a liver abscess, was used as the hypervirulence control.

### Statistical analysis

Statistical analysis was conducted using SPSS 26.0 (IBM Corp., Armonk, NY, USA), employing Student’s *t*-test to assess the growth rate, *in vitro* competition, and survival in serum between ST307 and ST11 isolates. Statistical significance was set at *P* < 0.05. Additionally, survival curves of *G. mellonella* infected with ST11 and ST307 isolates were subjected to analysis using the log-rank test.

## Data Availability

Genome sequences of the strains used in this study have been deposited in the GenBank database under BioProject number PRJNA1012333.

## SUPPLEMENTAL MATERIAL

Supplemental material msystems.01369-23-s0001.doc Tables S1-S3; Figure S1.

ASM does not own the copyrights to Supplemental Material that may be linked to, or accessed through, an article. The authors have granted ASM a non-exclusive, world-wide license to publish the Supplemental Material files. Please contact the corresponding author directly for reuse.
