# Supplementary material for: Outbreak of NDM-5-producing Klebsiella pneumoniae ST307: an emerging high-risk antimicrobial resistance clone in Shanghai, China
Source: mSystems. 2024 Mar 20;9(4):e01369-23. doi: 10.1128/msystems.01369-23 (PMC11019902; doi:10.1128/msystems.01369-23)
Supplement: Supplemental material — Tables S1-S3; Figure S1. [file msystems.01369-23-s0001.doc]

**Table S1 Clinical history of nine patients with NDM-5-producing ST307 CRKP isolates**

| Isolates | Age/sex | Isolation date | Specimen | Ward (patients type) | Hospital days | Antibiotic treatment | Invasive Procedures | Underlying diseases | Outcomes |
| --- | --- | --- | --- | --- | --- | --- | --- | --- | --- |
| Kp173 | 61y/M | 9/5/2022 | CVC | EICU (emergency) | 20days | SCF, MEM, TZP, MXF, IPM | CVC, BMA | NK/T-cell lymphoma of the nasal cavity | Dead |
| Kp178 | 86y/F | 9/10/2022 | Blood | EICU (emergency) | 12days | FEP | Thoracocentesis, BMA | Heart failure | Improvement |
| Kp181 | 57y/F | 9/12/2022 | Sputum | EICU (rheumatology) | 12days | MEM, MXF, SCF, SMZ, COL, TGC, VAN | CVC, catheter, TI, Bronchoscopy | Dermatomyositis | Dead |
| Kp187 | 70y/M | 9/16/2022 | Secreta | Rheumatology ward | 31days | MFX, MEM | CVC, Thoracocentesis | Temporal arteritis | Discharged |
| Kp199 | 72y/M | 9/19/2022 | Sputum | EICU (rheumatology) | 23days | MEM, SMZ, SCF | CVC, catheter, TI, Bronchoscopy | Interstitial lung disease | Discharged |
| Kp216 | 71y/F | 9/27/2022 | Urine | EICU (emergency) | 22days | TGC, MEM | None | Diabetes | Discharged |
| Kp249 | 85y/M | 10/28/2022 | Sputum | EICU (emergency) | 63days | MEM, FEP, LEV, TGC, MNO | CVC, Bronchoscopy, Thoracocentesis | Fever | Dead |
| Kp266 | 64y/M | 11/10/2022 | Blood | EICU (rheumatology) | 28days | IPM, TZP, MNO | BMA; catheter; TI | Adult-onset Still's disease | Discharged |
| Kp299 | 41y/F | 12/6/2022 | Urine | Rheumatology ward | 26days | FEP, AMK | Thoracic puncture | Systemic Lupus Erythematosus | Discharged |

M male; F female; CVC central venous catheter; EICU emergency intensive care unit; SCF [cefperazone sulbactam](../../../../D:/%25E6%259C%2589%25E9%2581%2593%25E8%25AF%258D%25E5%2585%25B8/Dict/8.10.3.0/resultui/html/index.html#/javascript:;); MEM meropenem; TZP piperacillin tazobactam;

MXF [moxifloxacin](../../../../D:/%25E6%259C%2589%25E9%2581%2593%25E8%25AF%258D%25E5%2585%25B8/Dict/8.10.3.0/resultui/html/index.html#/javascript:;); IPM imipenem; FEP [cefepime](../../../../D:/%25E6%259C%2589%25E9%2581%2593%25E8%25AF%258D%25E5%2585%25B8/Dict/8.10.3.0/resultui/html/index.html#/javascript:;); SMZ Sulfamethoxazole; COL colistin; TGC tigecycline; VAN vancomycin; LEV [levofloxacin](../../../../D:/%25E6%259C%2589%25E9%2581%2593%25E8%25AF%258D%25E5%2585%25B8/Dict/8.10.3.0/resultui/html/index.html#/javascript:;); MNO minocycline; CXM cefuroxime;

AMK [amikacin](../../../../D:/%25E6%259C%2589%25E9%2581%2593%25E8%25AF%258D%25E5%2585%25B8/Dict/8.10.3.0/resultui/html/index.html#/javascript:;); BMA bone marrow aspiration; TI tracheal intubation;

**Table S2 Genomic characteristics of Kp178**

| Genome ID | Bases (bp) | Type | Inc type | Resistance determinants |
| --- | --- | --- | --- | --- |
| Chromosome | 5375566 | circle | Not appilicable | *bla*_SHV-28_, *OqxA, OqxB* |
| pKp178-NDM-5 | 45403 | circle | IncX3 | *bla*_NDM-5_ |
| pKp178-CTX-M-15 | 79990 | circle | Not typed | *bla*_LAP-2_, *qnrS1*, *dfrA14* |
| pKp178-DHA-1 | 206048 | circle | IncFIB(pNDM-Mar) | *bla*_DHA-1_ |
| pKp178-qnrB1 | 230274 | circle | IncFIB(K), IncFII(K) | *qnrB1*, *sul2*, *tet(A)* |
| pA | 12798 | circle | ColRNAI | None |
| pB | 3852 | circle | Not typed | None |
| pC | 2877 | linear | Not typed | None |

**Table S3 Genetic background of the three ST11 isolates selected for fitness and virulence comparison**

| Isolates | K | ST type | Resistance determinants |
| --- | --- | --- | --- |
| Kp94 | KL64 | ST11 | *bla*_KPC-2_, *bla*_CTX-M-65_, *qnrS1*, *LAP2*, *bla*_TEM-1D_, *bla*_SHV-11_, *drfA14* |
| Kp144 | KL64 | ST11 | *bla*_KPC-2_, *bla*_CTX-M-65_, *qnrS1*, *LAP2*, *bla*_TEM-1D_, *bla*_SHV-11_, *drfA14* |
| Kp257 | KL64 | ST11 | *bla*_KPC-2_, *bla*_CTX-M-65_, *rmtB*, *qnrS1*, *LAP2*, *bla*_TEM-1D_, *drfA14* |


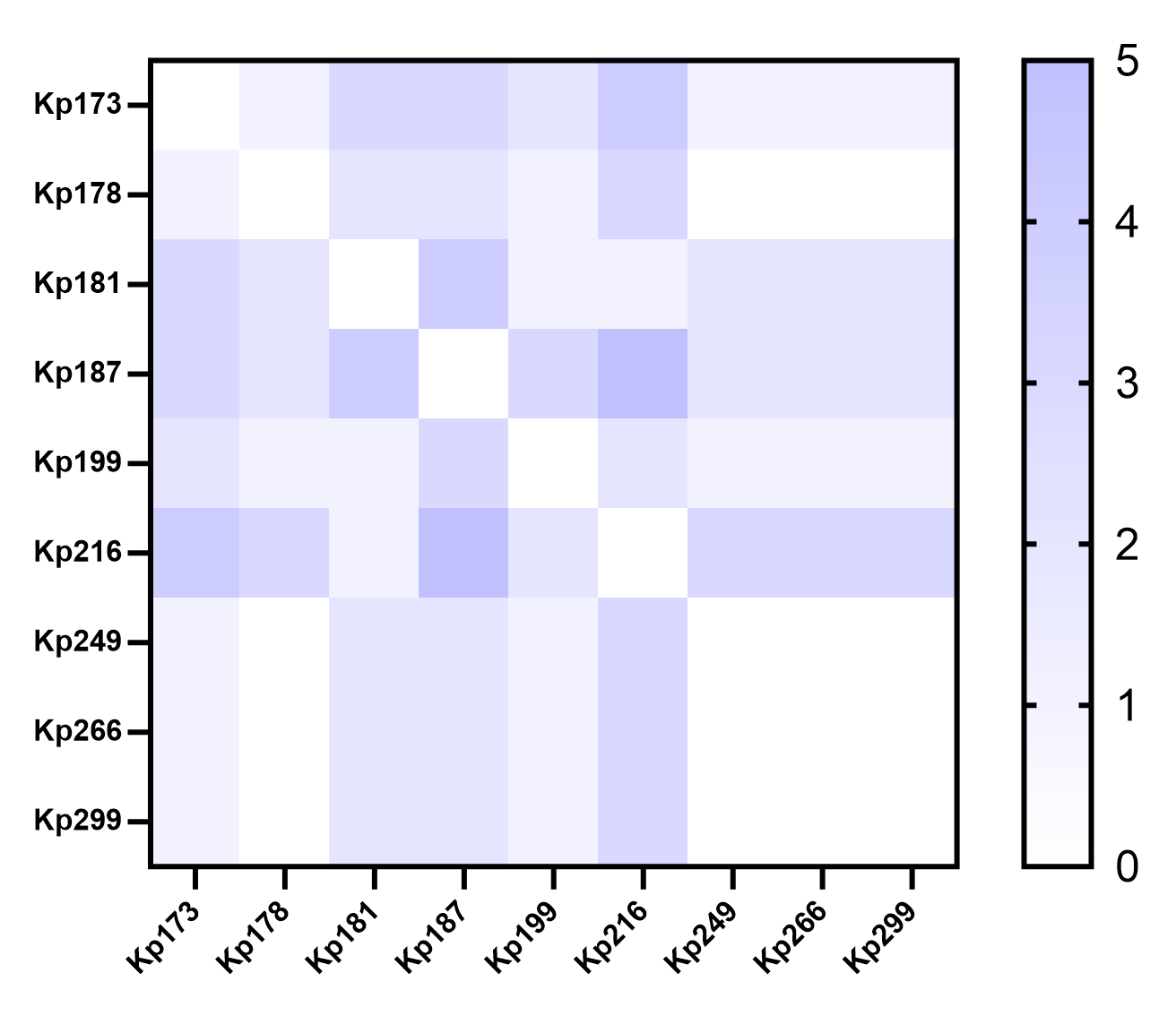


**Figure S1: Paired SNP distance of ST307 *K. pneumoniae* isolates.**
